# MRTF-A通过HOTAIR调控非小细胞肺癌细胞A549的增殖及迁移

**DOI:** 10.3779/j.issn.1009-3419.2019.02.02

**Published:** 2019-02-20

**Authors:** 琨 张, 渝斌 周, 刚 冯, 富春 曾

**Affiliations:** 610072 成都，四川省人民医院胸外科 Department of Thoracic Surgery, Sichuan Provincial People's Hospital, Chengdu 610072, China

**Keywords:** 肺肿瘤, MRTF-A, HOTAIR, 增殖, 迁移, Lung neoplasms, A549, MRTF-A, HOTAIR, Proliferation, Migration

## Abstract

**背景与目的:**

非小细胞肺癌（non-small cell lung cancer, NSCLC）作为肺癌的一种，由于其高发病率一直备受关注。准确揭示其发病机制对于NSCLC的诊断以及治疗具有重要的指导意义。MATF-A作为转录调控因子在多种肿瘤的发生发展过程中发挥着重要作用，可以调控多种肿瘤细胞的迁移过程。HOTAIR是近年研究发现的一个长链非编码RNA，在多种肿瘤中异常表达并参与多种肿瘤的增殖及迁移的进程。本研究旨在探究MRTF-A通过HOTAIR调控NSCLC的增殖及迁移进程。

**方法:**

构建MRTF-A的过表达质粒及干扰质粒，检测对A549细胞增殖及迁移的影响。设计合成HOTAIR的siRNA检测对A549细胞增殖及迁移的作用。qRT-PCR检测MRTF-A对HOTAIR表达的调控作用。构建HOTAIR的启动子检测MRTF-A对HOTAIR启动子活性的影响。

**结果:**

过表达MRTF-A促进A549细胞的增殖，沉默MRTF-A的表达抑制A549细胞的增殖及迁移。干扰HOTAIR的表达抑制A549细胞的增殖及迁移。MRTF-A能够影响HOTAIR的表达，同时能够调控HOTAIR启动子的活性。

**结论:**

MRTF-A通过HOTAIR调控NSCLCA549细胞的增殖及迁移进程。

## Abstract (en)

**Background and objective:**

Non-small cell lung cancer (NSCLC) is a kind of lung cancer, because its high incidence has been concerned. Therefore, it has great significance to reveal the pathogenesis of NSCLC. As a transcriptional regulatory factor, MATF-A plays an important role in the development of multiple tumors, can regulate the migration process of a variety of tumor cells. HOTAIR is a long non-coding RNA (LncRNA) found in recent years, which expresses abnormally in multiple tumors and is involved in the proliferation and migration of multiple tumors. The aim of this study is to explore the role of MRTF-A through HOTAIR to regulate the proliferation and migration of NSCLC cell A549 cell.

**Methods:**

We constructed the overexpression plasmid and interfering plasmid of MRTF-A, and detected the effect of MRTF-A on the proliferation and migration of A549 cells by CCK8 and wound healing methods respectively. Then, we designed the siRNA of HOTAIR to detect its effect on the proliferation and migration of A549 cells. Through qRT-PCR, we detected the effect of MRTF-A on HOTAIR expression. Finally, we constructed HOTAIR's promoter, and detect the effect of MRTF-A on HOTAIR promoter activity by luciferase reporter gene test.

**Results:**

Overexpression of MRTF-A promotes the proliferation and migration of A549 cells, while silent MRTF-A inhibits its proliferation and migration. Next, we found that interfered HOTAIR expression inhibited the proliferation of A549 cells. We found that MRTF-A could influence the expression of HOTAIR and regulate the activity of HOTAIR promoter.

**Conclusion:**

MRTF-A regulates the proliferation and migration of A549 cell through HOTAIR.

非小细胞肺癌（non-small cell lung cancer, NSCLC）是肺癌的主要类型之一，占肺癌的80%-85%，在全球范围内具有较高的发病率以及死亡率^[1, 2]^。由于早期不具备明显的临床症状，患者发现时已经错过最佳治疗时期，致使其5年生存率不足15%^[3, 4]^。尽管诸多治疗手段不断推广应用，但由于NSCLC的迁移以及侵袭特性，患者长期生存率及生存状况不尽人意^[5, 6]^。

MRTF-A是转录调控因子SRF的辅助因子。Rho信号通路中MRTF-A与G-actin结合阻滞了其核运输，抑制了靶基因的激活。MRTF-A通过与SRF形成转录调控复合体，结合到靶基因的启动子区域调控基因的表达^[7-9]^。有文献^[10, 11]^报道，在乳腺癌中MRTF-A能够调控基质金属蛋白酶的表达（MMP3及MMP9）进而调控乳腺癌MCF-7细胞的迁移。

LncRNA是近年研究发现的一类长度超过200 nt非编码RNA，在真核细胞内普遍表达但是不编码蛋白质^[12]^。大量研究证明，lncRNA能够通过与蛋白质、DNA或RNA相互作用，在表观遗传水平、转录水平和转录后水平调控基因的表达，进而参与细胞增殖、凋亡、迁移以及侵袭等生物学进程^[13, 14]^。HOTAIR是2007年发现的一个LncRNA，在多种肿瘤细胞中存在异常表达，其异常表达与肿瘤的发生发展迁移以及侵袭密切相关^[15, 16]^。因此，准确揭示MRTF-A与HOTAIR在NSCLC发生发展以及迁移侵袭中的作用具有重要的临床意义。本文拟通过实验研究MRTF-A及HOTAIR对NSCLC增殖迁移的影响以及MRTF-A对HOTAIR表达的作用。

## 1 材料与方法

### 1.1 材料

RPMI-1640培养基、胎牛血清购于美国Gibco公司。HOTAIR的siRNA购于中国广州锐博生物科技有限公司。MRTF-A抗体购Abcam公司。Odyssey双色红外激光成像系统购于美国LICOR公司。CCK8购于Sigma公司。Trizol、SYBR及Lipo2000购于美国Invitrogen公司。RevertAid First Strand cDNA Synthesis Kit购自加拿大Fermentas公司。肺癌细胞株A549由本实验室保存。

### 1.2 细胞培养

肺癌细胞A549培养于含10%FBS的1640培养基中，培养液含青霉素/链霉素100 U/mL，将细胞置于37 ℃、5%CO_2_培养箱中培养。

### 1.3 细胞转染

取对数生长期A549肺癌细胞，并于转染前24 h接种至6孔板（3×10^5^个/孔）继续培养，待细胞生长融合度达70%-80%时进行转染。转染依照Lipo2000说明书，将质粒（siRNA）转染入A549细胞中，在37 ℃及5%CO_2_培养箱中继续培养48h，用于后续相关实验检测。

### 1.4 实时荧光定量PCR

Trizol法提取NSCLC细胞A549/DDP的总RNA，利用RevertAid First逆转录试剂盒得到cDNA，以cDNA为模板，分别以MRTF-A、GAPDH及HOTAIR引物进行qRT-PCR反应。MRTF-A上游引物：5’-ACCGTGACCAATAAGAATGC-3’，下游引物：5’-CCGCTCTGAATGAGAATGTC-3’。HOTAIR上游引物：5’-TAGGCAAATGTCAGAGGGTT-3’，下游引物：5’-ACACAAGTAGCAGGGAAAGG-3’。GAPDH引物，上游引物：5’-CGAG ATCCCTCCAAAATCAA-3’，下游引物：5’-TTCACACCCATG AC GAACAT-3’。

### 1.5 Western blot检测蛋白表达水平

收集细胞，用细胞裂解液RIPA裂解提取总蛋白。SDS-PAGE凝胶电泳分离，恒流300 mA转移至PVDF印迹膜。5%脱脂牛奶封闭1 h后，加入一抗，4 ℃孵育过夜。次日PBS洗膜，二抗室温孵育1 h。用化学发光法显色，Odyssey双色红外激光成像系统。

### 1.6 CCK8检测细胞增殖

取对数生长期的A549细胞，以0.3×10^5^个/mL接种于96孔板中100 μL/孔，置于37 ℃、5%CO_2_培养箱中培养12 h。Lipo2000转染质粒（siRNA）48 h，将旧培养基吸去，置换含10%CCK-8的新鲜培养基，37 ℃、5%CO_2_继续培养3 h，测450 nm吸光度值，计算细胞的增殖率。

### 1.7 Transwell实验检测细胞迁移

取转染后细胞，调整细胞浓度为1×10^5^个/mL，分别将300 μL细胞悬液和700 μL含10%胎牛血清的RPMI-1640加入每Transwell上室和下室，并于37℃、5%CO_2_条件下培养48 h；取出Transwell小室，吸弃液体，医用棉签擦净内部基底膜；PBS漂洗，4%低聚甲醛固定10 min；结晶紫染液染色20 min；清水漂洗，显微镜下观察并拍照。

### 1.8 统计学分析

实验数据使用SPSS 17.0（SPSS, Chicago, USA）软件处理，各组实验数据以均数±标准差（Mean±SD）表示。组间统计学差异显著性比较采用*t*检验，单因素*ANOVA*分析，*P* < 0.05表示有统计学意义，本文中每个独立实验重复次数*n*≥3。

## 2 结果

### 2.1 MRTF-A促进A549细胞的增殖及迁移

在A549细胞中转染MRTF-A的过表达质粒之后，MRTF-A的表达量显著增高，是对照组的18倍（图 1A、图 1B）。通过CCK8实验检测细胞增殖率变化，通过Transwell小室迁移实验检测对A549细胞迁移影响，结果如图 1所示。该结果表明过表达MRTF-A显著促进A549细胞的增殖。在72 h时，过表达MRTF-A细胞增殖是对照组的1.5倍（图 1C）。与对照组相比过表达MRTF-A之后，A549细胞的迁移能力加强，细胞迁移率是对照组的3.5倍（图 1D、图 1E）。为进一步验证MRTF-A能够调控A549细胞的增殖及迁移，我们在A549细胞中转染MRTF-A的干扰质粒如图 1F所示，与对照组相比转染shMRTF-A质粒之后，MRTF-A的表达量下调55%。同样的结果在蛋白水平得到验证（图 1G）。转染shMRTF-A之后，在72h检测A549细胞的增殖率是对照组的60%（图 1H）。通过迁移实验证实转染shMRTF-A质粒之后，A549细胞的迁移率是对照组的55%（图 1I、图 1J）。由此可以得出MRTF-A能够调控A549细胞的增殖及迁移的结论。

**1:**
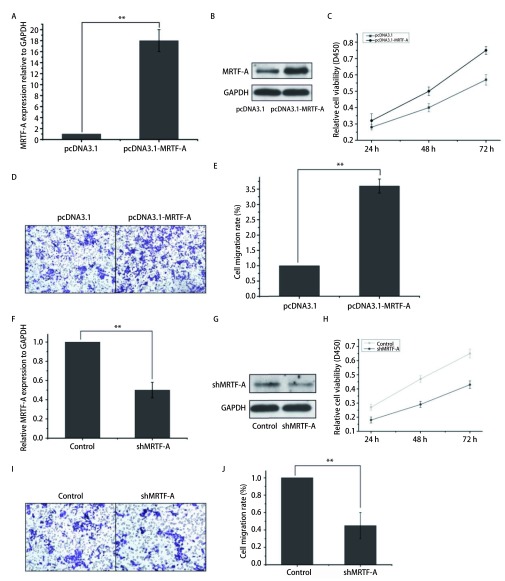
过表达MRTF-A促进A549细胞的增殖及迁移。A：过表达MRTF-A后的qRT-PCR验证；B：过表达MRTF-A后的Western blot验证；C：CCK8实验检测细胞增殖；D：transwell实验检测细胞迁移；E：过表达细胞与对照细胞迁移率的柱形图比较（^**^P < 0.01）；F :干扰MRTF-A后的qRT-PCR验证；G：干扰MRTF-A后的Western blot验证；H：CCK8实验检测细胞增殖；I：Transwell实验检测细胞迁移；J：干扰细胞与对照细胞迁移率的柱形图比较（^**^P < 0.01） Overexpression of MRTF-A promotes proliferation and migration ofA549 cells. A: qRT-PCR verification after overexpression of MRTF-A; B: Western blot verification after overexpression of MRTF-A; C: CCK8 assay for detection of cell proliferation; D: transwell assay for cell migration; E: bar graph of overexpressing cells versus control cell mobility (^**^P < 0.01); F: qRT-PCR validation after MRTF-A interference; G: Western blot validation after MRTF-A interference; H: CCK8 assay for cell proliferation; I: Transwell assay for cell migration; J: bar graph of interference cells versus control cell mobility (^**^P < 0.01)

### 2.2 沉默HOTAIR的表达抑制A549细胞的增殖及迁移

为验证LncRNA HOTAIR对A549细胞增殖及迁移的影响，我们设计合成了HOTAIR的siRNA，将siRNA的阴性对照（NC-siRNA）及siRNA-HOTAIR转染进入A549细胞，通过qRT-PCR检测HOTAIR的干扰效率（图 2A）。A549细胞转染siRNA-HOTAIR之后细胞增殖速度显著下降。对照组在72 h时增殖率是实验组的1.8倍（图 2B）。沉默HOTAIR表达，A549细胞的迁移率降低。与对照组相比，转染siRNA-HOTAIR的实验组迁移率是对照组的1/5（图 2C、图 2D）。由此可以得出HOTAIR能够调控A549细胞增殖及迁移的结论。

**2:**
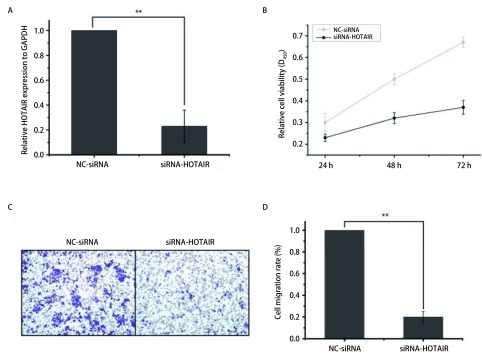
沉默HOTAIR抑制A549细胞的增殖及迁移。A：沉默HOTAIR后的qRT-PCR验证；B：CCK8实验检测细胞增殖；C：Transwell实验检测细胞迁移；D：沉默HOTAIR组与对照组细胞迁移率的柱形图比较（^**^P < 0.01） Silent HOTAIR expression inhibited proliferation and migration of A549 cells. A: qRT-PCR validation after HOTAIR silent; B: CCK8 assay for cell proliferation; C: Transwell assay for cell migration; D: bar graph of silent cells versus control cell mobility (^**^P < 0.01)

### 2.3 MRTF-A调控HOTAIR的表达

为验证MRTF-A是否能够调控HOTAIR的表达，我们在A549细胞中转染MRTF-A的过表达质粒及干扰质粒检测HOTAIR的表达量的变化，结果如图 3所示。在A549细胞中转染MRTF-A的过表达质粒，MRTF-A的表达量是对照组的13倍。与对照组相比，HOTAIR的表达量显著上升是对照组的2.7倍（图 3A）。A549细胞中转染MRTF-A的干扰质粒，MRTF-A的表达量显著下降是对照组的50%。与对照组相比，转染shMRTF-A之后HOTAIR的表达量明显下降是对照组的60%（图 3B）。由此可以得出MRTF-A调控HOTAIR表达的结论。

**3:**
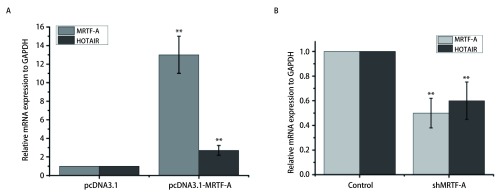
MRTF-A调控HOTAIR的表达。A：qRT-PCR检测过表达MRTF-A后HOTAIR的mRNA水平变化；B：qRT-PCR检测干扰MRTF-A后HOTAIR的mRNA水平变化 MRTF-A regulates HOTAIR expression. A: qRT-PCR detects changes in HOTAIR mRNA levels after overexpression of MRTF-A; B: qRT-PCR detects changes in HOTAIR mRNA levels after interference with MRTF-A

### 2.4 MRTF-A调控HOTAIR启动子活性的变化

为进一步验证MRTF-A对HOTAIR的调控作用，查阅MRTF-A相关文献发现MRTF-A能够与CArG-box序列特异性结合。通过UCSC网站在HOTAIR启动子上游680 bp位置找到一个CArG-box序列类似序列，构建了HOTAIR的启动子。在A549细胞中转染MRTF-A的过表达质粒及干扰质粒检测对HOTAIR启动子活性的影响，结果如图 4所示。A549细胞中转染MRTF-A的过表达质粒及HOTAIR启动子质粒48 h之后，与对照组相比HOTAIR的启动子活性上调5倍（图 4A）。与之相反，在A549细胞中转染MRTF-A的干扰质粒及HOTAIR的启动子质粒之后，HOTAIR启动子活性下降45%（图 4B）。由此可以得出MRTF-A能够调控HOTAIR启动子活性的结论。

**4:**
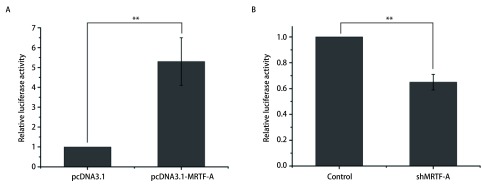
MRTF-A调控HOTAIR启动子活性。A：过表达MRTF-A后HOTAIR启动子活性的比较（^**^P < 0.01）；B:干扰MRTF-A后HOTAIR启动子活性的比较（^**^P < 0.01） MRTF-A regulates HOTAIR promoter activity. A: Comparison of HOTAIR promoter activity after overexpression of MRTF-A (^**^P < 0.01); B: Comparison of HOTAIR promoter activity after interference with MRTF-A (^**^P < 0.01)

### 2.5 沉默HOTAIR的表达可以rescue过表达MRTF-A对A549增殖和迁移的促进作用

为验证MRIF-A对肺癌细胞A549的增殖和迁移的促进作用是否与调控HOTAIR有关，共转染pcDNA3.1-MRIF-A和HOTAIR的siRNA，通过qRT-PCR验证转染效果，结果显示转染pcDNA3.1-MRIF-A后MRIF-A的表达量明显增高且HOTAIR的表达量也增高，同时转染pcDNA3.1-MRIF-A和HOTAIR的siRNA后，HOTAIR的表达量有所下降，说明转染成功（图 5A）。应用CCK8及Transwell实验检测共转染后对细胞增殖和迁移的影响，结果表明过表达pcDNA3.1-MRIF-A促进细胞增殖，但是共转染pcDNA3.1-MRIF-A和HOTAIR的siRNA后细胞增殖减慢（图 5B）。Transwell的结果表明共转染pcDNA3.1-MRIF-A和HOTAIR的siRNA后，细胞的迁移率较过表达MRIF-A组减慢（图 5C、图 5D）。结果说明了共转染pcDNA3.1-MRIF-A和HOTAIR的siRNA可以rescue过表达MRTF-A对A549增殖和迁移的促进作用。

**5:**
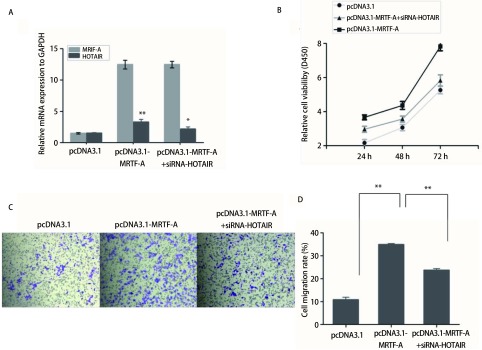
共转染pcDNA3.1-MRIF-A和HOTAIR的siRNA对细胞增殖和迁移的影响。A：qRT-PCR验证转染效果（^**^P < 0.01, ^*^P < 0.05）；B：CCK8实验检测细胞增殖；C:transwell实验检测细胞迁移；D：三组细胞迁移率的比较（^**^P < 0.01） Effect of co-transfected with pcDNA3.1-MRIF-A and siRNAs- HOTAIR on cell proliferation and migration. A: qRT-PCR verified transfection efficiency (^**^P < 0.01, ^*^P < 0.05); B: CCK8 assay for cell proliferation; C: transwell assay for cell migration; D: comparison of three groups of cell migration (^**^ *P* < 0.01)

## 3 讨论

肺癌作为一种高死亡率的肿瘤，究其发病的原因涉及到多个方面。原癌基因的激活、抑癌基因失活、基因突变、DNA甲基化以及非编码RNA的异常表达等原因均会导致肿瘤发生，而肺癌5年生存率偏低与肿瘤的迁移密切相关^[17-19]^。因此准确揭示肺癌的发生发展及迁移的机制对于肺癌的诊断以及治疗具有重要的指导意义。

Rho信号通路中，RhoC及其激酶ROCK通过调控细胞骨架组建参与肿瘤的迁移进程。在调控细胞骨架构建过程中，Rho效应激酶通过G-actin影响MRTFs（MRTF-A/MRTF-B）的出入核控制其与靶基因的结合，进而调控靶基因的表达^[20-22]^。MRTF-A作为血清反应因子SRF的辅助因子通过与SRF形成MRTF-A-SRF转录调控复合体结合到靶基因启动子区域的CArG-box序列上调控基因的表达^[23, 24]^。在乳腺癌肿瘤MDA-MB-231细胞中，Rho-actin-MRTF-A-SRF信号通路能够调控肿瘤细胞的迁移与侵袭^[25]^。此外，有文献报道MRTF-A通过调节某些基因的转录，在细胞生长、分化、迁移及肌生成过程中发挥重要作用^[26]^。本研究数据表明，在NSCLC中MRTF-A能够调控A549细胞的增殖及迁移。

HOX反义基因（HOTAIR）是近年发现的一个长链非编码RNA，通过调控基因表达及染色质动力学在各种癌症中发挥着重要的作用。研究发现，HOTAIR通过组蛋白甲基化转移酶PRC2及组蛋白去甲基化酶LSD1沉默基因的表达^[13, 15, 27]^。据文献报道，HOTAIR在乳腺癌、肺癌、卵巢癌、肝癌、结直肠癌、胰腺癌、食管鳞状癌及恶性胶质瘤中均存在异常表达，并在这些肿瘤的增殖及迁移进程中发挥重要作用^[28]^。HOTAIR能够通过调节一系列基因的表达参与调控细胞周期进程、肿瘤的增殖、EMT进程、肿瘤的迁移及侵袭进程^[29]^。我们的研究表明，HOTAIR能够调控NSCLC A549细胞的增殖及迁移的进程。

综上，本研究通过在A549细胞中转染MRTF-A及shMRTF-A质粒证实MRTF-A调控A549细胞增殖及迁移的结论。通过沉默A549细胞中HOTAIR的表达，证明HOTAIR在A549细胞增殖及迁移的过程中发挥重要作用。A549细胞中转染MRTF-A及shMRTF-A质粒得出MRTF-A影响HOTAIR表达及其启动子活性的结果。由此推测，MRTF-A-SRF-HOTAIR信号通路在NSCLC增殖及迁移的过程中可能发挥着重要的作用，其具体的分子作用机制还有待进一步探究，能否将HOTAIR作为NSCLC诊断及治疗的靶点仍需进一步探索。
